# Reconfigurable Nonlinear Activation Using Low‐Loss Ge_3_Sb_2_Te_15_ for Photonic Neural Networks

**DOI:** 10.1002/nap2.70212

**Published:** 2026-07-14

**Authors:** Yida Dong, Zhuoxuan Zhu, Yifan Zhu, Lichong Zhao, Lei Lei, Zihan Geng, Yida Li, X.‐D. Xiang, Heshan Yu, Mei Shen

**Affiliations:** ^1^ Southern University of Science and Technology Shenzhen China; ^2^ Tianjin University Tianjin China; ^3^ Shenzhen Polytechnic University Shenzhen China; ^4^ Shenzhen University Shenzhen China; ^5^ Tsinghua Shenzhen International Graduate School Shenzhen China

**Keywords:** Ge–Sb–Te system, integrated silicon photonics, optical phase‐change material (OPCM), reconfigurable non‐linear activation function

## Abstract

The escalating demand for high‐performance computing has spurred interest in integrated silicon photonics to overcome the constraints of traditional von Neumann architectures. Reconfigurable photonic devices with low optical loss, high‐speed switching, and nonvolatile operation are essential especially for photonic neural networks. This study introduces a robust high‐throughput methodology that serves as a primary engine for accelerating compositional search within the ternary Ge–Sb–Te (GST) system. Through this approach, we unveiled Ge_3_Sb_2_Te_15_ (GST3215), a novel optical phase‐change material (OPCM) that mitigates the high optical losses inherent in conventional OPCMs like Ge_2_Sb_2_Te_5_ (GST225). With reduced extinction coefficients and an improved figure of merit, GST3215 seamlessly integrates into hybrid silicon micro‐ring resonator (MRR), achieving lower insertion loss, a broader modulation range, and precise multilevel optical modulation—ideal traits for reconfigurable photonic systems. Moreover, GST3215 supports programmable nonlinear activation functions (e.g., ReLU, ELU, RBF) in a photonic neural network, lifting classification accuracy on the FashionMNIST dataset from 84.5% to 87.7%. These findings position GST3215 as a game‐changer for large‐scale photonic integration and optical computing.

## Graphical abstract

Composition‐engineered, low‐loss Ge_3_Sb_2_Te_15_ is identified from high‐throughput GST spreads and integrated with silicon microring resonators. Multistate optical programming produces tunable resonance responses and diverse fixed‐wavelength optical nonlinear activation functions, connecting phase‐change material discovery with programmable nanophotonic devices for reconfigurable photonic neural‐network computing.

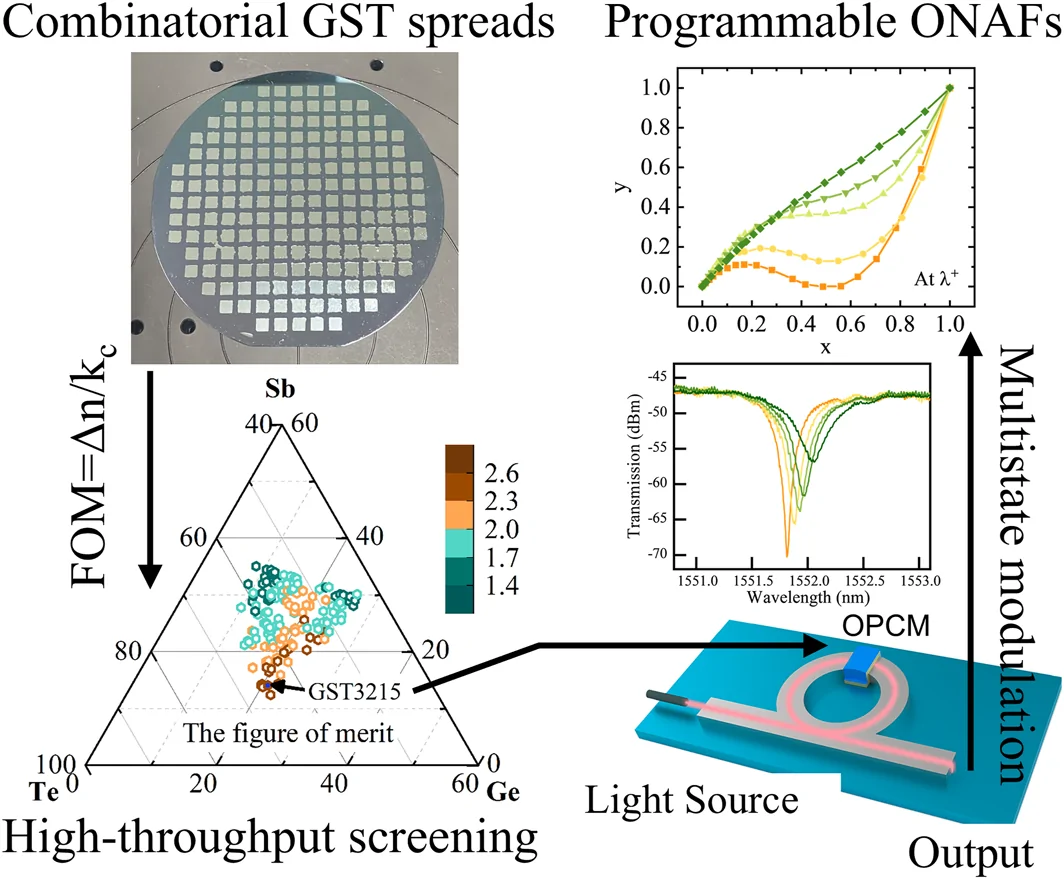

## 1 Introduction

The rapid proliferation of data‐intensive applications has exposed the inefficiencies of traditional von Neumann architectures, including memory bottlenecks and high energy consumption [1, 2]. These shortcomings have fueled interest in alternative paradigms with integrated silicon photonics emerging as a promising candidate due to its high bandwidth, parallelism, and rapid processing capabilities, which offers a path to overcome the constraints of traditional electronic frameworks [3, 4, 5, 6, 7, 8]. However, realizing large‐scale photonic systems requires reconfigurable photonic devices that deliver low optical loss, high‐speed switching, and nonvolatile operation [9, 10]. These attributes are especially crucial for applications like photonic neural networks, which rely on programmable nonlinear activation functions to perform complex tasks such as pattern recognition and decision‐making [11, 12].

A critical challenge in this domain is the development of optical phase‐change materials (OPCMs) capable of modulating light efficiently with minimal loss [13, 14, 15]. Within the ternary Ge–Sb–Te (GST) family, very limited compositions have been explored so far, materials such as Ge_2_Sb_2_Te_5_ (GST225) offer significant refractive index contrast, reliable cycling endurance, nonvolatile switching and rapid switching speeds, making them suitable for photonic applications [16, 17, 18, 19, 20, 21]. However, their high optical losses in the crystalline state hinder performance [22], particularly in photonic neural networks where nonlinear activation functions—such as rectified linear units (ReLU) [23], exponential linear units (ELU) [24], and radial basis functions (RBF) [25]—depend on optical resonators or interferometers, and material‐induced losses degrade signal integrity and device quality factor (Q‐factor).

To address this challenge, we developed a high‐throughput methodology to accelerate compositional exploration within the GST system. This approach enabled the systematic synthesis and characterization of GST thin‐film composition‐gradient spreads, culminating in the discovery of Ge_3_Sb_2_Te_15_ (GST3215), a novel OPCM with reduced optical loss and enhanced figure of merit (FOM) [26] compared to conventional GST alloys. Integrated into hybrid silicon micro‐ring resonators (MRRs), GST3215 delivers lower insertion loss, a broader modulation range, and precise multilevel modulation, making it ideal for reconfigurable photonic systems. Additionally, its nonvolatile properties facilitate programmable nonlinear activation functions—such as ReLU, ELU, and RBF—boosting photonic neural network accuracy on the FashionMNIST dataset from 84.5% to 87.7%. These advancements position GST3215 as a transformative material for large‐scale photonic integration and optical computing with the high‐throughput methodology serving as a critical enabler.

## 2 Results and Discussion

Amorphous GST thin‐film composition‐gradient spreads were fabricated by co‐sputtering onto 3‐inch silicon wafers at room temperature using two different target configurations (Ge/Sb/Te and GeTe/Sb/Te) to access distinct compositional regions. Detailed fabrication procedures are provided in the Methods section. Based on the compositions information determined by wavelength dispersive spectroscopy (WDS), the spread with the composition region closed to the Ge corner in the GST ternary phase diagram was named as Ge‐rich spread, whereas the spread having the composition region closed to the Te corner was named as Te‐rich spread (Supporting Information S1: Figure S1). The corresponding crystalline samples were subsequently obtained by thermal annealing following characterization in the amorphous state with the annealing conditions determined from temperature‐dependent ellipsometry measurements as described in the Methods Section 4.1.

We first systematically studied the structural and optical properties of the Ge‐rich spread by taking the Raman and ellipsometry measurements. With analyzing the Raman spectra of the whole Ge‐rich spread in crystalline state, four prominent peaks which center at ∼67, ∼110, ∼165, and ∼300 cm^−1^ corresponding to the A_1g_(1), *E*_g_(2), A_1g_(2), and ethane‐like Ge–Ge units, respectively, are determined. Then the Raman shift of the A_1g_(2) mode for the whole Ge‐rich spread are summarized in Figure 1a. The peak of A_1g_(2) mode related to the vibrations of defective octahedra moves to low Raman shift with increasing Te concentration. The similar change is also observed for the A_1g_(1) and *E*_g_(2) modes (Supporting Information S1: Figure S2). These indicate that the chemical bonding is weakened as the Te concentration raises. Meanwhile, the ethane‐like Ge‐Ge units are observed for almost half of samples on the Ge‐rich spread (Supporting Information S1: Figure S2), showing that doping more Ge elements can induce the formation of Ge‐Ge clustering in the GST matrix.

**FIGURE 1:**
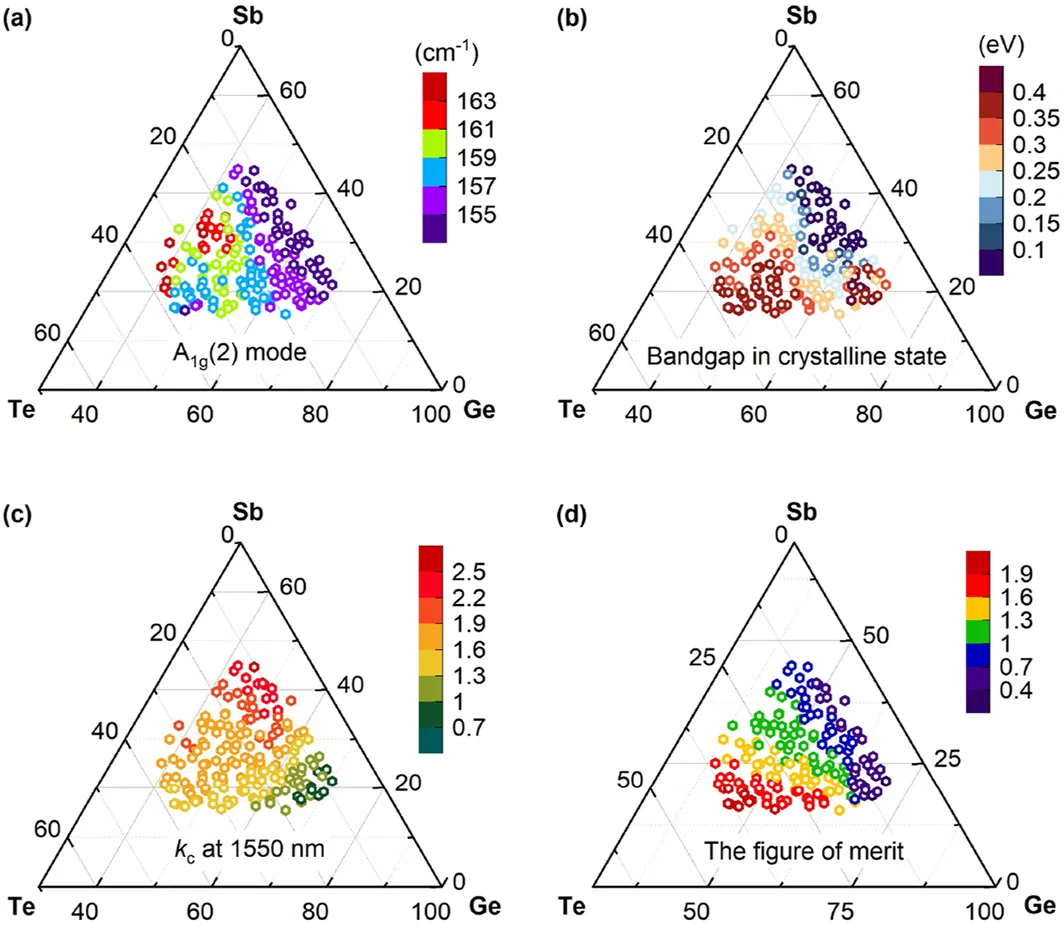
The structural and optical properties of the Ge‐rich spread. (a) The change in the Raman shift of A_1g_(2) mode. (b) The bandgap *E*_g_ in crystalline state. (c) The extinction coefficient kc in crystalline state. (d) The figure of merit Δ*n*/*k*_c_.

Afterward, the optical parameters are analyzed for the whole Ge‐rich spread at the wavelength of 1550 nm, which is a typical signal wavelength in integrated photonics. The bandgap in amorphous state is relatively large and also varies slightly with changing composition ratios (Supporting Information S1: Figure S3). However, the bandgap *E*_g_ in crystalline state is reduced significantly (Figure 1b), since the chemical bonding in crystalline state shows resonant feature, quite different from the covalent bonding in amorphous states [27, 28]. With increasing Te concentration, the weakening of the local chemical bonding of defective octahedra gives rise to the raising bandgap in the crystalline state. Meanwhile, the refractive index and extinction coefficient at the wavelength of 1550 nm are shown in Figure 1 and Supporting Information S1: Figure S3. Both the refractive index *n*_a_ and extinction coefficient *k*_a_ slightly varies with changing compositions in amorphous state (Supporting Information S1: Figure S3). Meanwhile, all the amorphous samples on the Ge‐rich spread have the low extinction coefficient *k*_a_. When GST thin‐film samples change into crystalline state, both the refractive index *n*_c_ and extinction coefficient *k*_c_ becomes larger compared to those in amorphous state (Figure 1c and Supporting Information S1: Figure S3). The extinction coefficient *k*_c_ is gradually reduced with decreasing Sb concentration, whereas the refractive index *n*_c_ raises when the Te concentration increases. Thereafter, the FOM is calculated using the equation Δ*n*/*k*_c_, where the Δn is the contrast in refractive index between the amorphous and crystalline states, and *k*_c_ is the extinction coefficient in crystalline state. The FOM shows the maximum for the thin‐film samples having the most Te concentration on this spread. Together with the observed decrease of *k*_c_ at lower Sb content, this trend indicates that reduced Sb content and increased Te content are favorable for improving the FOM within this compositional window. Unfortunately, the majority of the samples on this spread exhibit FOM values lower than GST225, with only a few demonstrating comparable performance [5].

Guided by the higher FOM observed toward the Te‐enriched region of the Ge‐rich spread, we further studied the Te‐rich spread to search for lower‐loss GST compositions. With analyzing the Raman spectra of the whole Te‐rich spread in crystalline state, three prominent modes A_1g_(1) (∼67 cm^−1^), *E*_g_(2) (∼110 cm^−1^), and A_1g_(2) (∼165 cm^−1^) are determined. The Raman shift of A_1g_(2) mode is reduced with raising Te concentration similar to that on the Ge‐rich spread (Figure 2a), whereas the *E*_g_(2) mode shifts to the higher Raman shift when Ge concentration decreases (Supporting Information S1: Figure S4). However, the mode of the ethane‐like Ge–Ge units centered at ∼300 cm^−1^ is not observed in the whole Te‐rich spread, indicating that there is no evident Ge impurity in these samples.

**FIGURE 2:**
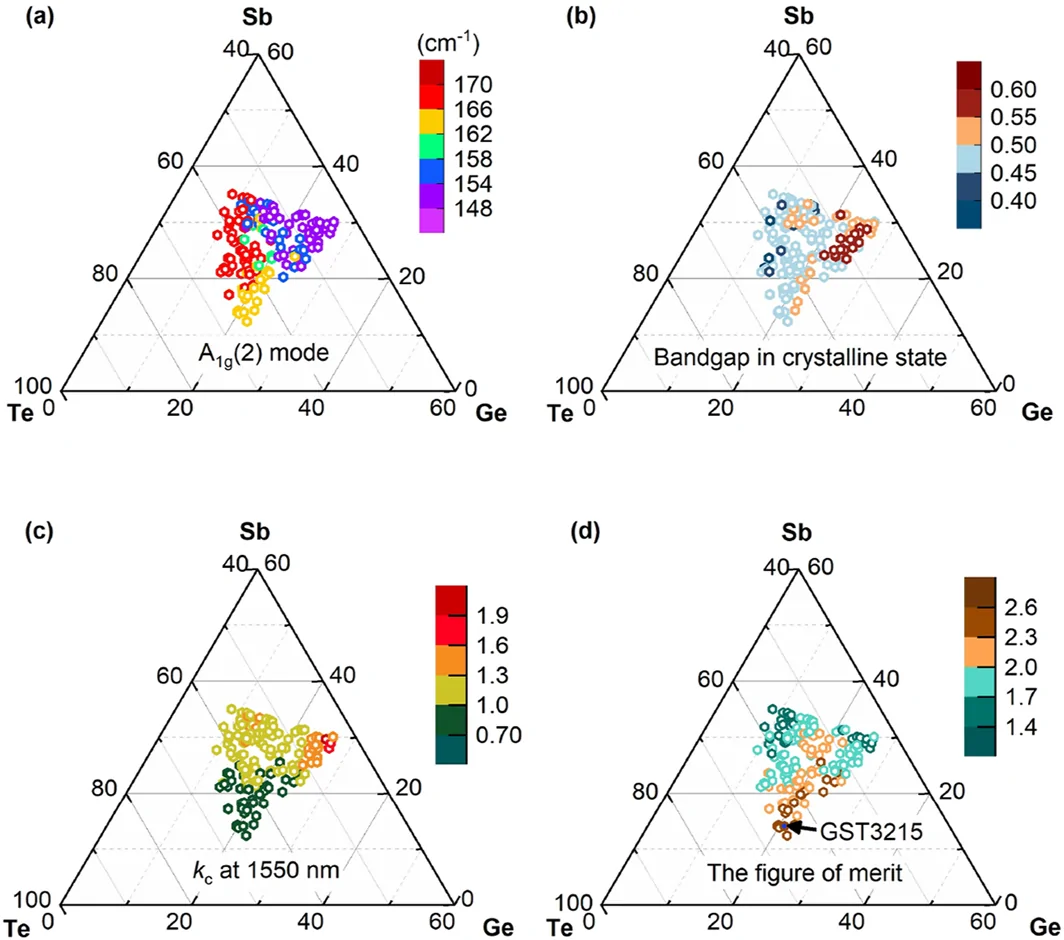
The structural and optical properties of samples on the Te‐rich spread. (a) The change in the Raman shift of A1g(2) mode. (b) The bandgap *E*_g_ in crystalline state. (c) The extinction coefficient kc in crystalline state. (d) The figure of merit Δ*n*/kc. The optimal composition is marked with blue solid hexagon.

Then the optical parameters are analyzed to explore some candidates for photonic applications. The bandgap for example in the crystalline state shows the maximum for the samples at the right corner of this composition region (Figure 2). With doping more Te atoms, the bandgap *E*_g_ is gradually reduced. These changes here are originating from the tuning of the resonant bonding in crystalline state, which is also able to change the refractive index *n*_c_ and extinction coefficient *k*_c_. All the amorphous samples have the low refractive index *n*_a_ and extinction coefficient *k*_a_ at the wavelength of 1550 nm (Supporting Information S1: Figure S4). For the crystalline samples, both the refractive index *n*_c_ and extinction coefficient *k*_c_ are reduced by increasing the Te concentration, in accordance with the change of bandgap for example in crystalline state. Within this compositional window, the region with higher Te content corresponds to lower Sb content, where *k*_c_ is correspondingly reduced, consistent with the trend observed in the Ge‐rich spread. Finally, the FOM for the whole spread is calculated using the equation Δ*n*/*k*_c_ to identify a novel OPCM for photonic applications. The FOM of all the samples on this Te‐rich spread are summarized in Figure 2d. The optimal sample whose FOM is superior to GST225 or other compositions on both Ge‐rich and Te‐rich spreads lies at the bottom corner of composition region of the Te‐rich spread. The composition of this sample is determined to be Ge3Sb2Te15 using the WDS, and henceforth, we refer to this novel OPCM as GST3215.

Compared to some reported OPCMs in the family of GST alloys, this novel OPCM GST3215 has some better optical properties in the infrared region [5, 28]. As shown in Figure 3, GST3215 has the low extinction coefficient in both amorphous and crystalline states, which can significantly optimize the loss in the photonic devices. At the wavelength of 1550 nm which is a typical signal wavelength in integrated photonics, the complex refractive indices (*n* + *ik*) of GST3215 are 3.79 + 0.02*i* in the amorphous state and 5.61 + 0.65*i* in the crystalline state. The full wavelength‐dependent optical constants of GST3215 in the amorphous state and after 200°C annealing are provided in Supporting Information S1: Figure S5 for independent verification. The extinction coefficient *k*_a_ of the amorphous GST3215 is almost zero. Meanwhile, the extinction coefficient *k*_c_ of GST3215 in crystalline state is much lower than that of some reported GST, for example, GST225, GST124, and GST214, etc. Thereafter, the FOM in the infrared region is calculated by using the formula Δ*n*/*k*_c_. Around the wavelength of 1550 nm, the novel OPCM GST3215 shows a much larger FOM than any reported GST alloys. Figure 3d illustrates Δ*n* as a function of *k*_c_ at 1550 nm, which facilitates a more detailed comparison between GST3215 and other reported GST alloys. In these GST alloys, GST3215 exhibits the lowest kc and moderate Δ*n*, further demonstrating its ability to maintain optical modulation capability while effectively reducing optical loss. The FOM of GST3215 at 1550 nm is 2.8, indicating our finding GST3215 is a good candidate in the integrated photonics.

**FIGURE 3:**
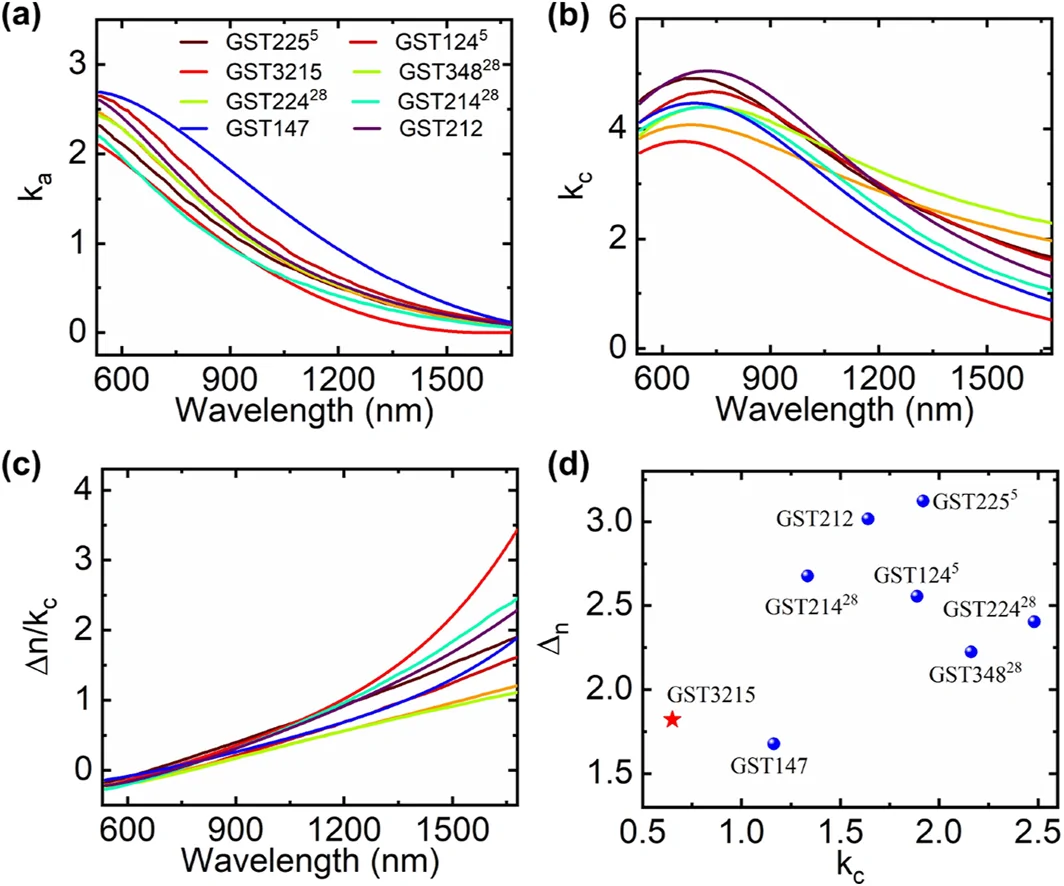
The comparison of optical properties between GST3215 and some other well‐known OPCMs (GST225 [5], GST124 [5], GST348 [28], GST224 [28] and GST214 [28]). (a) The extinction coefficient in amorphous state. (b) The extinction coefficient in crystalline state. (c) The figure of merit Δ*n*/*k*_c_, where Δ*n* is the contrast of refractive index between amorphous and crystalline states, and *k*_c_ is the extinction coefficient in crystalline state. (d) Δ*n* as a function of *k*_c_ at 1550 nm. The inset placed in panel (a) serves as a shared material‐label guide for panels (a–c).

Then, we fabricated GST3215‐MRRs to evaluate the performance of GST3215 as a tunable cladding material in integrated photonics. Figure 4a illustrates the structure of the GST3215‐MRR in which a 20 nm GST3215 thin film is deposited on the MRR. To prevent oxidation of GST3215 during the repeated phase transition, a 20 nm Al2O3 capping layer is applied atop the GST3215 film. The phase transitions of GST3215 were induced using a free‐space pulsed laser heat treatment system (PLHTS) [29] at 1064 nm, shown in Supporting Information S1: Figure S6b, employing a beam shaper to convert the Gaussian beam into a flat‐top distribution. The transmission spectra of both amorphous and crystalline GST3215 are recorded for various covering lengths ranging from 2 to 6 μm (Figure 4b). The extinction ratio (ER) variation reveals that a hybrid MRR with a 4 μm‐long GST3215 has the critical coupling.

**FIGURE 4:**
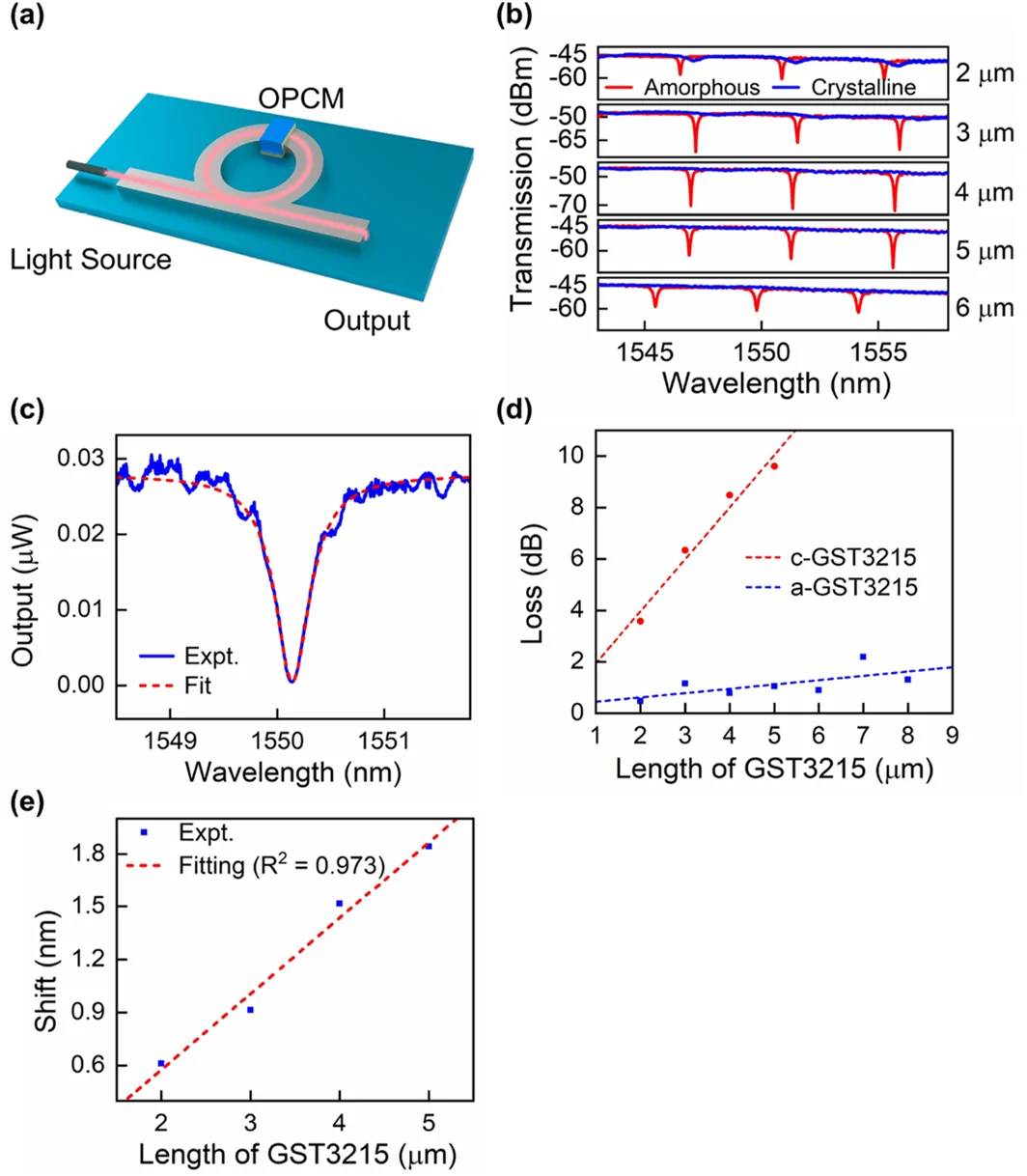
Transmission loss measurement of GST3215‐MRR. (a) The schematic of GST3215‐MRR (b) Transmission spectrum of ring resonators covered with varying lengths (2–6 μm) of GST3215. (c) Lorentzian fitting of the spectrum for the resonant dip. (d) Optical losses of amorphous GST3215 and crystalline GST3215 on MRR at various lengths. (e) Spectral shift from amorphous to crystalline with the increasing length of GST3215 on MRR.

Increasing the GST3215 length results in a broader resonance peak, indicating a corresponding rise in the optical loss. To quantify the optical modulation of GST3215 on MRRs, Lorentz fitting was applied to the transmission spectra near 1550 nm to extract the quality factor and also evaluate the loss using the Equation (1) [29]:

(1) $$\begin{array}{c} \text{Loss}=\frac{2\pi \lambda_{0}}{\text{FSR}}\left(\frac{1}{Q}-\frac{1}{Q_{0}}\right), \end{array}$$

where FSR is the free spectral range of MRR, *λ*_0_ is the resonant wavelength, *Q* and *Q*_0_ are the quality factors of the same MRR after and before the GST3215 deposition. A representative resonant peak and its Lorentzian fit (Figure 4c) demonstrates the high fidelity between experimental data and theoretical fitting. Loss measurements reveal a linear relationship between loss and GST3215 length in both amorphous and crystalline states as presented in Figure 4d. The attenuation coefficient *α* of GST3215 is 0.167 dB/μm in amorphous state and 2.023 dB/μm in crystalline state, yielding an attenuation modulation of 1.856 dB/μm. Simultaneously, a spectral shift of 0.429 nm/μm was observed during the phase transition, as shown in Figure 4e. Comparatively, the *α* of GST225 is 0.27 dB/μm in amorphous state and 7.60 dB/μm in crystalline state [30]. Through the adjustment and screening of the GST system, we successfully identified a formulation with lower optical absorption compared to the commonly used GST225.

The theoretical spectral shift (Δλ/L) can be estimated using the Equation (2) [31]:

(2) $$\begin{array}{c} \frac{\Delta \lambda }{L}\approx \frac{\Delta_{n\text{eff}}\lambda_{0}}{2\pi Rn_{neffa}}, \end{array}$$

where $\Delta n_{\text{eff}}$ is the effective index change caused by phase transition, and $n_{\text{effa}}$ is the real part of the effective index for the silicon waveguide covered by amorphous GST3215 (aGST3215). According to Equation (2) and the finite‐difference time‐domain (FDTD) simulation (see methods in Supporting Information S1: Figure S7), the theoretical $\Delta n_{\text{eff}}$ of GST3215 is 0.16, and spectral shift is 0.780 nm/μm. The real part of $\Delta n_{\text{eff}}$ is 0.088 higher than that of the 15 nm thick GST225 (∼0.074) [21]. These optimized optical properties, including enhanced absorption modulation and stable phase‐change modulation, indicate the potential of GST3215 for large‐scale photonic integration. A broader quantitative benchmark of GST3215‐MRRs and representative GST225‐integrated photonic devices is summarized in Supporting Information S1: Table S1, including programming conditions, Q factor, optical loss, modulation range, multilevel operation, and repeatability.

The reversible phase‐change characteristics and multilevel modulation capabilities of the photonic devices fabricated with GST3215 were further examined. Figure 5a shows the reset and set operations of a GST3215‐MRR with a 2 μm‐long GST3215, confirming its reversible tuning capability. A single 1 ns pulse with an energy density of 21.7 mJ/cm^2^ induces amorphization (Reset), whereas continuous pulses of 43.8 mJ/cm^2^ crystallize the GST3215 (Set). The resonance peak position and ER demonstrated good repeatability before and after the set and reset processes. We then conducted 10 repeated tests on the waveguide and extracted the extinction ratio of the resonance peak, demonstrating stable cycling performance (Figure 5b). An additional 1100‐cycle test on another GST3215‐MRR device further shows only a slight ER decrease and relatively stable amorphous‐state optical loss (Supporting Information S1: Figure S8). Figure 5c,d illustrate the transmission spectra of GST3215‐MRRs with 3 and 6 μm long GST3215 devices, respectively. For 3 μm GST3215‐MRR, single‐pulse energies ranging from 4.6 to 24.6 mJ/cm^2^ modulated the degree of amorphization, yielding 11 distinct extinction ratios. To achieve more distinct states, we divided the 6 μm long GST3215 into two regions and then processed them sequentially with energy ranging from 5.4 to 27.8 mJ/cm^2^ in a single pulse, successfully demonstrating 20 distinct states. Compared to bare MRR, we extract the loss from the multistate spectra and then analyzed the variation of $\Delta n_{\text{eff}}$ referring to the highest energy. The experimental values of loss and shift are fitted by using the Equations (1) and (2). After partitioning the longer GST3215‐MRR, more optical states could be achieved, demonstrating the feasibility of multilevel modulation for advanced photonic applications. Repeated multistate measurements on GST3215‐MRRs with a GST3215 coverage length of 3 μm and long‐term stability measurements provide further quantitative information on the reliability of GST3215‐MRRs (Supporting Information S1: Figure S8).

**FIGURE 5:**
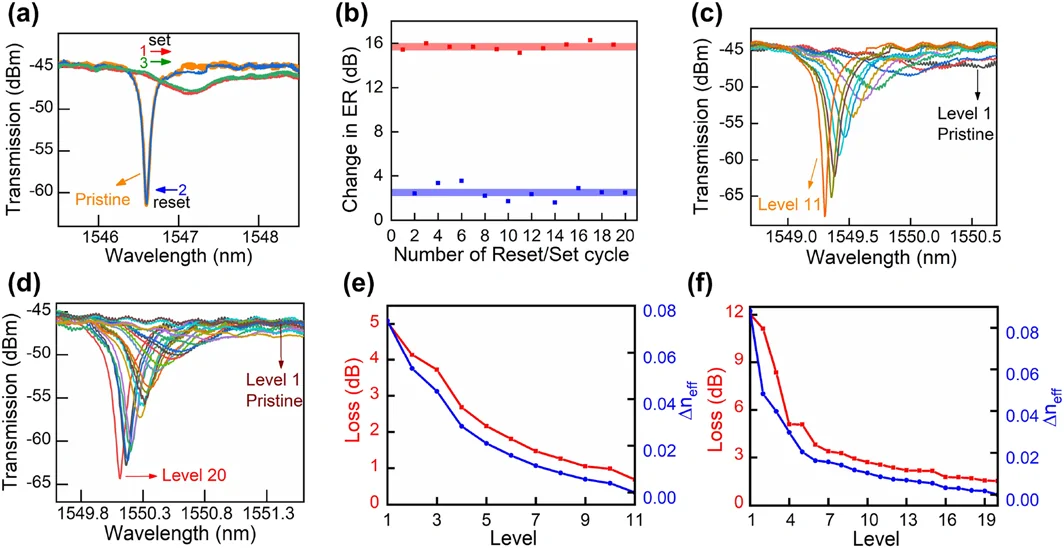
Multilevel optical switching and optical properties of GST3215‐MRR (a) SET and RESET operation. (b) The variation in the ER extracted from 10 cycles. (c) Multilevel optical switching of 3 μm GST3215‐MRR. (d) Multilevel optical switching of 6 μm GST3215‐MRR. Absorption loss and $\Delta n_{\text{eff}}$ of (e) 3 μm GST3215‐MRR and (f) 6 μm GST3215‐MRR.

Leveraging the optimized phase‐change material GST3215, we successfully implemented a reconfigurable ONAF (optical nonlinear activation function) unit exhibiting diverse nonlinear responses based on the MRR. Supporting Information S1: Figure S9 illustrates the experimental setup for nonlinear response measurements. Five intermediate states between the crystalline and amorphous phases were selected (Figure 6a). Meanwhile, the nonlinear responses were measured at three specific resonance wavelengths: *λ* (resonant wavelength) and *λ* ^±^ (*λ* ± 0.05 nm). To ensure a clear comparison of nonlinear behaviors across different material states, input, and output power values were normalized. The fixed‐wavelength nonlinear transmission originates from the combined effect of the programmed GST3215‐dependent resonance modulation and the power‐dependent nonlinear response of the silicon microring. In the silicon waveguide, Kerr‐induced self‐phase modulation introduces a power‐dependent nonlinear phase shift, whereas two‐photon absorption (TPA) and TPA‐induced free‐carrier absorption (FCA) introduce power‐dependent nonlinear loss. These power‐dependent phase and loss changes, together with the programmed GST3215‐dependent phase and attenuation, convert the input optical power into different nonlinear transmission responses at the selected fixed readout wavelength. The results in Figure 6 reveal that different readout wavelengths and programmed GST3215 states can generate distinct nonlinear activation behaviors, including ELU‐shaped, ReLU‐shaped, and RBF‐shaped ONAFs. The influence of the extinction ratio of the MRR for these behaviors have been explored. As the phase‐change material are crystallized under the laser excitation, the extinction ratio gradually decreased. At *λ*, the nonlinear activation function transitioned from ReLU to ELU characteristics, while at *λ* ^+^, the curvature of the RBF‐shaped ONAF progressively flattened toward linearity. Our findings demonstrate the tunable nonlinear response behaviors across both phase‐change material states and resonance wavelengths, offering a robust foundation for tailoring nonlinear activation functions.

**FIGURE 6:**
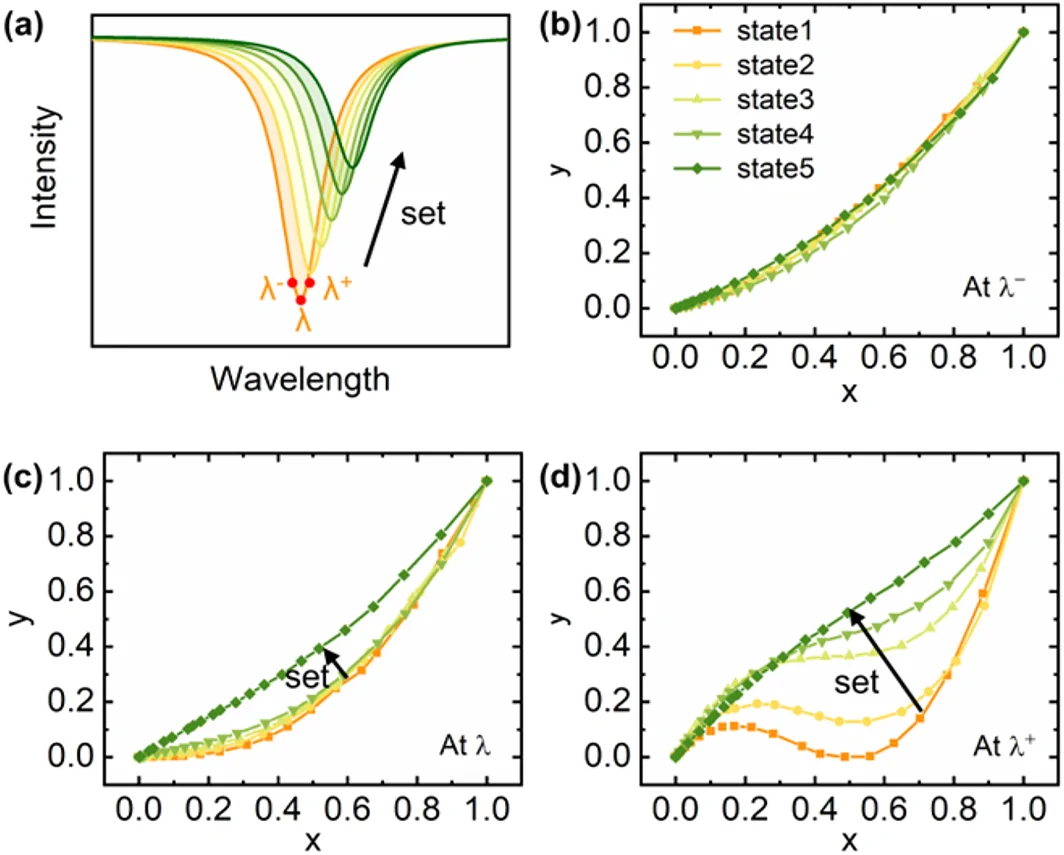
The schematic of nonlinear measurements. (a) The measurements at different states and wavelengths. (b–d) shows the non‐linear response at *λ*−, *λ* and *λ*+, respectively, where *x* and *y* represent the normalized input and output energy power, respectively.

To validate the applicability of our ONAFs from the measurement of nonlinearity of MRR, classification simulations were performed using a three‐layer fully connected neural network (Figure 7a). Raw input images were first flattened into one‐dimensional arrays and then fed to the input layer of neural network. In hidden layers, the neuron obtains the weighted summation of signals from all neurons in input layer and applies a nonlinear mapping to the summation where the nonlinear mapping behavior is controlled by activation function. The signal from output neuron presents the probability of category of the input image. Additionally, the nonlinear activation function hyperbolic tangent (Tanh) and linear transformation were tested as mathematical baselines. The cross‐entropy loss and recognition accuracy of the training and validation datasets during the training process are shown in Supporting Information S1: Figure S10. The corresponding final training/validation metrics for each experimentally fitted ONAF and each mathematical baseline are summarized in Supporting Information S1: Table S2. For the experimentally fitted ONAFs, the associated GST3215 state, readout wavelength, programming energy density, and state‐dependent optical‐loss metric are also included. The performance metrics of neural networks using the experimentally fitted ONAFs, Tanh, and linear transformation converge within 50 epochs. The performance of neural networks using the fitted ONAFs is close to that of the Tanh network and surpasses that of the network using only linear transformation. It is concluded that, compared with linear transformation, ONAFs enhance the neural networks' ability to learn complex mapping relationships from multidimensional data as well as the inference capability. Figure 7b illustrates the recognition accuracy of networks using ONAFs on the validation dataset, which offers more insight into the impact of different ONAFs on classification performance, including their ability to handle nonlinearity and gradient propagation. A neural network with an RBF‐shaped ONAF shows slightly lower performance on the validation dataset compared with ReLU‐ and ELU‐like ONAFs. Although RBF‐shaped ONAFs offer greater nonlinear flexibility due to their nonmonotonic nature, gradients approaching zero near extreme values can slow parameter updates and impede efficient gradient flow. In the present three‐layer fully connected network and FashionMNIST classification task, the localized response of the RBF‐shaped ONAF may make the network output more sensitive to the intermediate feature‐amplitude distribution, leading to slightly reduced validation performance. These results suggest that the suitability of a programmed ONAF should be evaluated together with the network architecture and task‐dependent feature distribution. The neural network achieving the highest classification accuracy utilizes ONAF‐ELU from state 3 at *λ* with classification accuracies of 90.5% on the training set and 87.7% on the validation set, surpassing the performance of the linear transformation network (86.5% and 84.5%, respectively). Figure 7c,d present the confusion matrices for the training and validation sets, respectively, providing detailed insights into the classification accuracy across different categories for refined analysis. To further evaluate the influence of cycling‐induced optical‐response fluctuations on the ONAF‐based neural‐network output, an ER/Q‐perturbation sensitivity analysis was performed and summarized in Supporting Information S1: Table S3. Overall, the network performs better on the training set than the validation set. Except for the “Shirt” category, the network demonstrates strong classification performance across the remaining nine categories with accuracies exceeding 80%. Notably, the classification accuracies for “Trouser” and “Bag” reach an impressive 96% in the validation set. The lower accuracy in the “Shirt” category can be attributed to significant misclassification as “T‐shirt.” Visually, “T‐shirt” and “Shirt” exhibit considerable similarities leading to overlapping features and subsequent misclassification. Further optimization of hyperparameters such as the number of layers, neuron counts, and fine‐tuning of activation functions, could enhance network performance and also reduce the classification errors.

**FIGURE 7:**
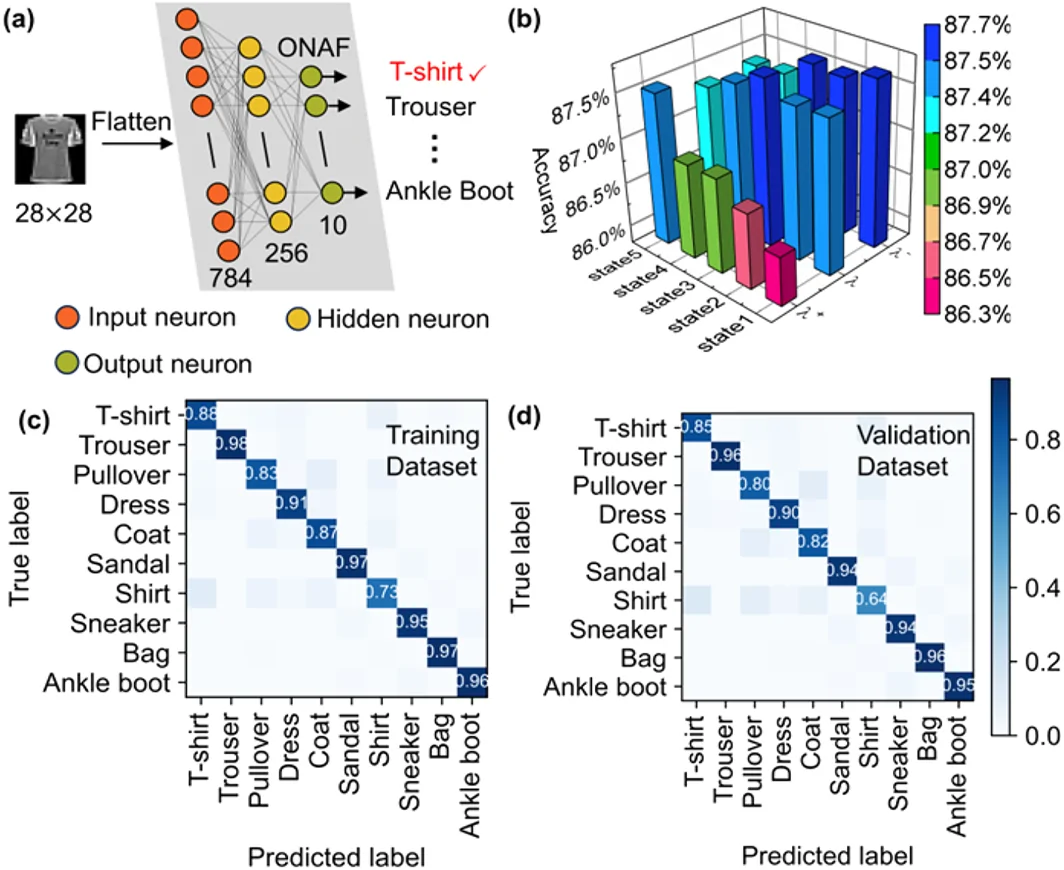
Neural network classification simulations. (a) The structure of three‐layer neural network. (b) The classification accuracy for all obtained ONAFs in validation dataset. (c) and (d) depict confusion matrix of neural network in training and validation datasets, respectively.

## 3 Conclusion

This study introduces Ge_3_Sb_2_Te_15_ (GST3215), a novel optical phase‐change material uncovered through a high‐throughput methodology that efficiently explored the ternary Ge‐Sb‐Te system. With an extinction coefficient of 0.02 (amorphous) and 0.65 (crystalline) and a FOM of 2.8 at 1550 nm, GST3215 surpasses conventional GST alloys such as GST225 in reducing optical losses. Integrated into hybrid silicon MRRs, it delivers low insertion loss (0.167 dB/μm amorphous, 2.023 dB/μm crystalline), broad modulation range, and stable multilevel operation up to 20 states. In optical nonlinear units, GST3215 enables programmable activation functions (ReLU, ELU, RBF), enhancing photonic neural network accuracy on the FashionMNIST dataset from 84.5% to 87.7%. GST3215 represents a significant advancement for large‐scale photonic integration and optical computing. By addressing critical material limitations and demonstrating practical utility in reconfigurable devices and neural networks, it paves the way for next‐generation photonic technologies. The high‐throughput methodology, serving as a robust engine for compositional discovery, underscores the potential for further OPCM innovations.

## 4 Methods

### 4.1 Preparation of GST Composition‐Gradient Spreads

GST composition‐gradient spreads were prepared by room‐temperature co‐sputtering onto 3‐inch Si wafers. To access different compositional regions in the GST ternary system, two types of spreads were fabricated using different target configurations. The Ge‐rich spread was prepared using Ge/Sb/Te targets, whereas the Te‐rich spread was prepared using GeTe/Sb/Te targets to more effectively shift the accessible compositional window toward the Te‐rich region.

The sputtering targets were arranged underneath the substrate with an angular spacing of approximately 120°. When the substrate holder angle was fixed during deposition, each target generated a thickness gradient along a different in‐plane direction. As a result, different positions on the wafer received different relative fluxes of the constituent species leading to a continuous compositional distribution across the wafer surface. During deposition, the Ar working pressure was maintained at 3 mTorr. The selected sputtering conditions produced continuous and uniform composition‐gradient films with sufficiently smooth surfaces for reliable WDS, Raman, and ellipsometry characterization.

Following characterization in the amorphous state, selected samples from the Ge‐rich spread were thermally annealed at 350°C, whereas selected samples from the Te‐rich spread were thermally annealed at 200°C to obtain the crystalline state. These annealing temperatures were selected based on temperature‐dependent ellipsometry measurements, as summarized in Supporting Information S1: Figure S11. Starting from 200°C, selected samples were annealed in 50°C intervals, and their optical constants were extracted after each annealing step. A significant change in the optical constants was used as an indicator of crystallization. For the Ge‐rich spread, although some positions showed optical‐constant changes below 350°C, 350°C was selected as the final annealing condition to ensure that the selected positions across the spread reached representative crystalline states for consistent composition‐dependent comparison. For the Te‐rich spread, clear optical‐constant changes were already observed at 200°C, and this temperature was therefore used as the representative crystalline‐state annealing condition.

### 4.2 The Raman and Ellipsometer Analysis

The structural and optical properties of Ge‐rich and Te‐rich spreads were collected by taking the Raman and ellipsometry measurements. The ellipsometry measurement were carried out using an ellipsometer from J. A. Woollam company. The incident angles were set as 55°, 65°, and 75° with the energy range of 0.7–3.1 eV. Afterward, the ellipsometer data were analyzed with the Tauc–Lorentz model to obtain the optical parameters, for example, refractive index *n*, and extinction coefficient *k*. [28] The Raman spectra were measured at room temperature over the range from 50 to 300 cm^−1^ for some spreads in the crystalline state and then were fitted using the Levenberg–Marquardt method. The normalized Raman spectra maps from the plotted sampling positions and representative Raman spectra for the Ge‐rich and Te‐rich composition‐gradient spreads are provided in Supporting Information S1: Figures S12 and S13, respectively, to supplement the extracted Raman peak‐position evolutions. Raman spectroscopy on the final annealed samples was used to assist in confirming the formation of representative crystalline states through the GST‐related crystalline modes. Meanwhile, the background of the Raman spectra was substituted with the Rampy, a Python library designed to assist with spectral data processing.

### 4.3 Device Fabrication and Testing

The fabrication of GST3215‐MRRs, as shown in Supporting Information S1: Figure S6a, begins with the definition of the MRR pattern on a Silicon‐On‐Insulator (SOI) substrate using electron‐beam lithography (EBL, Raith BV, EBPG 5150) with a positive‐tone resist (AR‐P 6200.09). The pattern was then transferred onto the silicon layer through inductively coupled plasma (ICP) etching (NAURA, GSE200Plus), defining the MRR structure with dimensions of 220 nm in thickness, 500 nm in width, and a 20 μm radius. Then, the GST3215 deposition region was defined using direct laser writing (DLW, Durham Magneto Optics, MicroWriter ML3) in conjunction with a double‐layer resist process utilizing LOR 5A and AZ5214. This method ensures the thorough removal of any residual photoresist and defines the area where the GST3215 will be deposited. The 20 nm GST3215 thin film was deposited onto the MRRs by magnetron co‐sputtering from GeTe, Sb, and Te targets to control the composition. The sputtering powers for the GeTe, Sb, and Te targets were set to 65, 13, and 62 W, respectively, under an Ar working pressure of 3 mTorr to obtain the target GST3215 composition. A 20 nm Al_2_O_3_ passivation layer was applied via atomic layer deposition (ALD) to prevent oxidation during phase transitions. The phase transitions of GST3215 were induced using a free‐space pulsed laser heat treatment system (PLHTS) [29] at 1064 nm. The transmission spectra of the GST3215‐MRRs were measured using a vertical coupling platform with a broadband light source, an optical spectrum analyzer (OSA, YOKOGAWA, AQ6370 B), and a polarization controller (Thorlabs, FPC562). Comparative analysis of the spectra from bare MRRs and GST3215‐MRRs enabled the evaluation of spectral shifts and optical losses induced by the GST3215.

The optical indices of 20 nm GST3215 thin film were characterized in a range from 400 to 1700 nm through ellipsometry before and after annealing for optical mode simulation. The fundamental quasi‐transversal electric (TE) mode distributions of silicon waveguides covered with GST3215 by at *λ* = 1550 nm were simulated by finite‐difference time‐domain (FDTD) method as shown in Supporting Information S1: Figure S7. The attenuation coefficients (*α*) of aGST3215 and cGST3215 are 0.04 dB/μm and 3.00 dB/μm, respectively.

### 4.4 Neural Network Classification Simulation

The classification simulations of FashionMNIST dataset based on pytorch which is a framework for building AI models were performed [32]. A three‐layer fully connected neural network has been built. The FashionMNIST dataset is used to train and validate the neural network which contains 70,000 different images from 10 categories. Then, the FashionMNIST dataset was divided into training and validation set which have 60,000 and 10,000 images, respectively. Each image in FashionMNIST is of 28 × 28 pixels. The batch size of dataset and epochs were set to 64 and 50, respectively. The parameters of the neural network were optimized by stochastic gradient descent (SGD) to reduce the cross‐entropy loss [33]. Classification accuracy is chosen as the performance metric for the neural network. To apply the experimentally measured nonlinear activation curves in classification simulations, mathematical expressions for these curves were required. Rational functions, where the numerator’s degree exceeds that of the denominator by one, were employed for curve fitting. All fitted nonlinear functions were subsequently used in the classification simulations alongside linear transformations and tanh for comparison.

## Author Contributions

**Yida Dong:** writing – review and editing, data curation, methodology, investigation, formal analysis, validation, visualization. **Zhuoxuan Zhu:** writing – original draft, data curation, writing – review and editing, methodology, investigation, visualization. **Yifan Zhu:** data curation. **Lichong Zhao:** software. **Lei Lei:** conceptualization. **Zihan Geng:** conceptualization. **Yida Li:** conceptualization. **X.‐D. Xiang:** conceptualization, funding acquisition. **Heshan Yu:** validation, data curation, investigation, conceptualization, funding acquisition. **Mei Shen:** writing – review and editing, resources, project administration, conceptualization, funding acquisition, validation, supervision.

## Funding

This work was financially supported by the Shenzhen Science and Technology Innovation Commission (Grants JCYJ20240813095259010, JCYJ20241202123919027, and JCYJ20220818100613028) and in part by the Tianjin Natural Science Foundation (Grant 23JCQNJC01680), and the National Natural Science Foundation of China (Grant 52402199).

## Conflicts of Interest

The authors declare no conflicts of interest.

## Supporting information


Supporting Information S1

## Data Availability

All data generated or analyzed during this study are included in this published article and its supplementary information files.
