# Supplementary material for: Reconfigurable Nonlinear Activation Using Low‐Loss Ge3Sb2Te15 for Photonic Neural Networks
Source: Nanophotonics. 2026 Jul 14;15(14):e70212. doi: 10.1002/nap2.70212 (PMC13642108; doi:10.1002/nap2.70212)
Supplement: Supplementary file 1 [file NAP2-15-e70212-s001.docx]

Supporting information:

**Reconfigurable Nonlinear Activation using Low-Loss Ge₃Sb₂Te₁₅ for Photonic Neural Networks**

Yida Dong,^†^ Zhuoxuan Zhu,^†^ Yifan Zhu, Lichong Zhao, Lei Lei, Zihan Geng, Yida Li, X.-D. Xiang,^*^ Heshan Yu,^*^ and Mei Shen^*^

1. **The composition information of the Ge-rich and the Te-rich spreads**

**Fig. S1**: The composition information of (a) the Ge-rich and (b) the Te-rich spreads.

2. **The structural information of the Ge-rich spread**

**Fig. S2:** The structural information of samples on the Ge-rich spread. (a) The A_1g_(1) mode, (b) the E_g_(2) mode, and (c) the ethane-like Ge-Ge units in crystalline state.

3. **The optical properties of the Ge-rich spread**

**Fig. S3:** The optical properties of samples on the Ge-rich spread. (a) The bandgap in amorphous state. (b) The extinction coefficient in amorphous state. The refractive index in (c) amorphous and (d) crystalline states.

4. **The structural and optical properties of the Te-rich composition-gradient spread**

**Fig. S4:** The structural and optical properties of the whole samples on the Te-rich composition-gradient spread. The Raman shift of (a) A_1g_(1) mode and (b) E_g_(2) mode. (c) The bandgap in amorphous state. The refractive index in (d) amorphous and (e) crystalline states; (f) The extinction coefficient in amorphous state.

5. **Optical constants of amorphous and 200℃ annealed GST3215**

**Fig. S5.** Wavelength-dependent optical constants of GST3215 in the amorphous state and after 200 °C annealing. The refractive index (n) and extinction coefficient (k) were extracted from spectroscopic ellipsometry measurements. The 200 °C annealed state corresponds to the representative crystalline state used for the Te-rich spread and GST3215 optical-property analysis.

6. **Fabrication processes of GST3215-MRR**

**Fig. S6:** (a) Diagrams illustrating the fabrication steps for the GST3215-MRR. (b) A schematic representation of the PLHTS used to trigger phase transitions in the GST3215 on the MRR.

7. **Finite Difference Time Domain (FDTD) simulation of GST3215**

**Fig. S7:** Simulated fundamental TE mode profiles of (a) aGST3215 and (b) cGST3215.

8. **Benchmarking of GST3215-MRR with representative GST225-integrated photonic devices**

Table S1. Benchmarking of GST3215-MRRs against representative GST225-integrated photonic devices.

| Ref. | Material / platform | Programming method | Energy / power metric | Q factor | Loss / insertion loss | Modulation / shift | Multilevel / repeatability |
| --- | --- | --- | --- | --- | --- | --- | --- |
| This work | GST3215/  Si MRR | 1064 nm Free-space PLHTS | Reset: 21.7 mJ/cm², 1 ns; Set: 43.8 mJ/cm², 200 ns per pulse | Bare/a/c: 7100/5873/2615 for 2μm | a/c: 0.167/2.023 dB/μm | 0.429 nm/μm shift; ER up to 24 dB in multilevel modulation | 20 states; 1100 cycles |
| Zheng et al.^1^ | GST225/ Si MRR | 532 nm free-space pulsed laser | Reset: 31 mJ/cm²; Set: 10 mJ/cm² per pulse | - | a/c: 0.27/7.6 dB/μm | 0.70 nm/μm phase modulation; ER up to 33 dB | Quasi-continuous tuning |
| Rudé et al.^2^ | GST225/ Si racetrack resonator | 975 nm free-space pulsed laser | Reset: 45 mW, 20 ns; Set: 12 mW, 300 ns | Bare/a/c/re-a: 6528/5656/3641/5411 | On-state IL: 2.5 dB | 12.36 dB modulation; 168 pm shift | - |
| Ríos et al.^3^ | GST225/ Si_3_N_4_ waveguide/ring | On-chip guided optical pulses | 8-level operation: 372-601 pJ; lowest binary switching energy: 13.4 pJ | - | - | 21% readout change; >10 dB WDM modulation | Up to 8 levels; 100 cycles with ±7.1% confidence interval; retention at least 3 months |
| Faneca et al.^4^ | GST225/ Si_3_N_4_ waveguide/ring | Hot-plate annealing; FEM thermal simulation | FEM: 15 mW, 20 ns | Bare/a/c: 1.4 × 10^4^/3869/1938 | a/c: 0.039/2.86 dB/μm | ER: 18 dB | - |

9. **Extended reliability characterization of GST3215-MRRs**

Extended cycling, multistate repeatability, and long-term stability measurements of GST3215-MRRs are summarized in Fig. S8. The extended 1100-cycle test was performed on another GST3215-MRR device. During the cycling test, the ER shows only a slight decrease, while the amorphous-state optical loss remains relatively stable, supporting repeatable optical modulation during extended phase-transition cycling. The crystalline-state loss is not plotted because the crystalline-state resonance dips are relatively shallow and broad, which introduces larger uncertainty in the extraction of crystalline-state optical loss. SEM observation after cycling reveals local surface damage around the processed region, suggesting that the slight ER degradation is likely related to local damage of the passivation layer induced by repeated laser processing.

Repeated multistate measurements were further performed on GST3215-MRRs with a GST3215 coverage length of 3 μm under the same multistate programming conditions, and the corresponding error bars were calculated from the repeated measurements. For long-term stability evaluation, a GST3215-MRR was measured shortly after fabrication and again after approximately two years. The device could still be programmed to a comparable amorphous-state extinction ratio, although the Q factor decreased from 4071 to 3048. This degradation is likely associated with device aging during the two-year interval, because the device was not protected by optimized packaging or long-term encapsulation.

**Fig. S8:** Reliability measurements of GST3215-MRRs. (a) Evolution of the Q factor during the extended 1100-cycle test. (b) Evolution of the extinction ratio during the extended 1100-cycle test. (c) Amorphous-state optical-loss variation during the extended 1100-cycle test. (d) Repeated multistate loss modulation measurements on GST3215-MRRs with a GST3215 coverage length of 3 μm, with error bars representing the standard deviation. (e) Transmission spectra of a GST3215-MRR measured shortly after fabrication and after approximately two years. (f) SEM image of the GST3215-MRR after the extended 1100-cycle test.

10. **Experimental setup for nonlinear response measurements**

**Fig. S9:** Experimental setup for nonlinear response measurements. TL, tunable laser. PM, power meter. VOA, variable optical attenuator. PC, polarization controller.

11. **Metrics of neural networks for training and validation datasets during training process**

**Fig. S10:** (a) and (b) show the loss and accuracy of networks for training dataset during training process. (c) and (d) show the loss and accuracy of networks for testing dataset during training process.

12. **Device-level cost and neural-network performance**

For each experimentally fitted ONAF, the GST3215 state, readout wavelength, ONAF curve shape, programming energy density, ER, Q factor or extracted optical-loss metric, final training loss, final validation loss, training accuracy, and validation accuracy are summarized. For the mathematical baselines, including Tanh and linear transformation, the final training/validation metrics are listed for comparison, while the device-level programming and optical-loss metrics are marked as not applicable. This table complements Fig. 7b by providing the complete numerical training/validation metrics and correlating the performance of each experimentally fitted ONAF with its corresponding device-level cost.

Table S2. Device-level cost and neural-network performance of experimentally fitted GST3215-MRR ONAFs and mathematical baselines.

| GST3215 state | Programming energy density (mJ/cm^2^) | ER (dB) | Q factor | Optical loss (dB/$\boldsymbol{\mu m}$) | Readout wavelength | ONAF shape | Training loss | Training accuracy | Validation loss | Validation accuracy |
| --- | --- | --- | --- | --- | --- | --- | --- | --- | --- | --- |
| State1 | 23.3 | 22.9 | 4703 | 0.189 | 1551.771 | ELU-shaped | 0.28252 | 0.90127 | 0.35167 | 0.87669 |
|  |  |  |  |  | 1551.821 | ReLU-shaped | 0.27101 | 0.90572 | 0.35709 | 0.8751 |
|  |  |  |  |  | 1551.871 | RBF-shaped | 0.29299 | 0.90104 | 0.39162 | 0.86346 |
| State2 | 19.7 | 18.7 | 3629 | 0.292 | 1551.827 | ELU-shaped | 0.28416 | 0.89954 | 0.35273 | 0.8756 |
|  |  |  |  |  | 1551.877 | ReLU-shaped | 0.27224 | 0.90515 | 0.35041 | 0.875 |
|  |  |  |  |  | 1551.927 | RBF-shaped | 0.29469 | 0.89879 | 0.38317 | 0.86644 |
| State3 | 17.9 | 16.7 | 3492 | 0.310 | 1551.877 | ELU-shaped | 0.28067 | 0.90179 | 0.35046 | 0.8759 |
|  |  |  |  |  | 1551.927 | ReLU-shaped | 0.27164 | 0.9048 | 0.35037 | 0.87669 |
|  |  |  |  |  | 1551.977 | RBF-shaped | 0.28965 | 0.89992 | 0.36562 | 0.86863 |
| State4 | 16.1 | 14.7 | 3243 | 0.346 | 1551.917 | ELU-shaped | 0.28231 | 0.90089 | 0.3541 | 0.87371 |
|  |  |  |  |  | 1551.967 | ELU-shaped | 0.27692 | 0.90332 | 0.3536 | 0.8749 |
|  |  |  |  |  | 1552.017 | RBF-shaped | 0.29947 | 0.89571 | 0.36934 | 0.86863 |
| State5 | 12.5 | 9.5 | 2989 | 0.389 | 1552.001 | ELU-shaped | 0.28899 | 0.8981 | 0.35722 | 0.87351 |
|  |  |  |  |  | 1552.051 | ELU-shaped | 0.29305 | 0.89737 | 0.35908 | 0.87331 |
|  |  |  |  |  | 1552.101 | RBF-shaped | 0.29071 | 0.89799 | 0.35412 | 0.875 |
| Tanh |  |  |  |  |  |  | 0.29994 | 0.89432 | 0.35786 | 0.874 |
| Linear |  |  |  |  |  |  | 0.39014 | 0.86524 | 0.43777 | 0.84544 |

13. **Sensitivity analysis of ONAF performance under cycling-induced ER/Q variations**

To quantify the influence of cycling-induced optical-response fluctuations on the ONAF-based neural-network output, the ER and Q-factor variations extracted from the 1100-cycle measurement were introduced into the ONAF model. The extracted ER and Q factor were 11.15 ± 1.03 dB and 2102.64 ± 81.66, respectively. Because the absolute ER and Q values of the cycling-test spectra are not necessarily identical to those of the state-1 spectrum used for ONAF construction, the relative fluctuations were used as perturbation amplitudes. The corresponding relative variations were approximately 9.21% for ER and 3.88% for Q factor.

Based on these relative variations, three ONAF responses were constructed for each fixed readout wavelength: the nominal ONAF obtained from the unperturbed state-1 optical response, the low-ER/Q ONAF obtained by decreasing ER and Q by one relative standard deviation, and the high-ER/Q ONAF obtained by increasing ER and Q by one relative standard deviation. For each readout wavelength, the same three-layer fully connected neural network used in the main text was first trained using the nominal ONAF. The trained weights were then fixed, and only the activation function was replaced by the corresponding low- and high-ER/Q perturbed ONAFs. This fixed-weight sensitivity analysis was used to evaluate whether the experimentally observed cycling-induced ER/Q fluctuations would significantly affect the neural-network output.

Table S3. Recognition-accuracy variation under ER/Q-perturbed ONAFs at different fixed readout wavelengths.

| Fixed readout wavelength | Nominal ONAF accuracy | Low-ER/Q ONAF accuracy | High-ER/Q ONAF accuracy | Perturbed accuracy, mean ± s.d. | Worst-case change |
| --- | --- | --- | --- | --- | --- |
| λ_0_ − 0.05 nm | 88.46% | 88.34% | 88.48% | 88.41 ± 0.10% | −0.12 pp |
| λ_0_ | 87.83% | 87.65% | 86.75% | 87.20 ± 0.64% | −1.08 pp |
| λ_0_ + 0.05 nm | 88.76% | 87.41% | 86.71% | 87.06 ± 0.50% | −2.05 pp |

Here, “pp” denotes percentage points. The results show that the influence of ER/Q fluctuation is readout-wavelength dependent. In particular, the λ_0_ − 0.05 nm readout point exhibits only a −0.12 percentage-point worst-case accuracy change, indicating that the ONAF-based network output can remain stable under the experimentally observed cycling-induced optical-response fluctuation when an appropriate fixed readout wavelength is selected.

14. **Temperature-dependent ellipsometric characterization of representative Ge-rich GST samples**

**Fig. S11:** Temperature-dependent optical constants of representative samples on the Ge-rich composition-gradient spread. (a) A representative sample showing an abrupt increase in optical constants at 350 °C. (b) A representative sample showing optical-constant changes below 350 °C followed by relatively stable optical constants upon further annealing. Colors denote annealing temperatures, while solid and dashed lines represent *n* and *k*, respectively.

15. **Normalized Raman spectra of the Ge-rich and Te-rich composition-gradient spreads**

**Fig. S12:** Normalized Raman spectra of the Ge-rich composition-gradient spread. (a) Normalized Raman spectra map collected from the plotted sampling positions on the Ge-rich spread, where the horizontal axis represents the Raman shift, the vertical axis represents the sampling-position index, and the color scale represents the normalized Raman intensity. (b-d) Representative Raman spectra selected from different compositional regions of the Ge-rich spread.

**Fig. S13:** Normalized Raman spectra of the Te-rich composition-gradient spread. (a) Normalized Raman spectra map collected from the plotted sampling positions on the Te-rich spread, where the horizontal axis represents the Raman shift, the vertical axis represents the sampling-position index, and the color scale represents the normalized Raman intensity. (b-d) Representative Raman spectra selected from different compositional regions of the Te-rich spread.

Reference:

(1) Zheng, J.; Khanolkar, A.; Xu, P.; Colburn, S.; Deshmukh, S.; Myers, J.; Frantz, J.; Pop, E.; Hendrickson, J.; Doylend, J.; et al. GST-on-silicon hybrid nanophotonic integrated circuits: a non-volatile quasi-continuously reprogrammable platform. *Optical Materials Express* **2018**, *8* (6). DOI: 10.1364/ome.8.001551.

(2) Rudé, M.; Pello, J.; Simpson, R. E.; Osmond, J.; Roelkens, G.; van der Tol, J. J. G. M.; Pruneri, V. Optical switching at 1.55 μm in silicon racetrack resonators using phase change materials. *Applied Physics Letters* **2013**, *103* (14). DOI: 10.1063/1.4824714 (acccessed 6/13/2026).

(3) Ríos, C.; Stegmaier, M.; Hosseini, P.; Wang, D.; Scherer, T.; Wright, C. D.; Bhaskaran, H.; Pernice, W. H. P. Integrated all-photonic non-volatile multi-level memory. *Nature Photonics* **2015**, *9* (11), 725-732. DOI: 10.1038/nphoton.2015.182.

(4) Faneca, J.; Garcia-Cuevas Carrillo, S.; Gemo, E.; de Galarreta, C. R.; Domínguez Bucio, T.; Gardes, F. Y.; Bhaskaran, H.; Pernice, W. H. P.; Wright, C. D.; Baldycheva, A. Performance characteristics of phase-change integrated silicon nitride photonic devices in the O and C telecommunications bands. *Optical Materials Express* **2020**, *10* (8), 1778-1791. DOI: 10.1364/OME.397833.
